# Emerging Evidence of Thresholds for Beneficial Effects from Vitamin D Supplementation

**DOI:** 10.3390/nu10050561

**Published:** 2018-05-03

**Authors:** Robert Scragg

**Affiliations:** School of Population Health, University of Auckland, Private Bag 92019, Auckland 1142, New Zealand; r.scragg@auckland.ac.nz; Tel.: +64-9-923-6336

**Keywords:** dose–response, randomized controlled trials, thresholds, vitamin D supplementation

## Abstract

Publications from clinical trials of vitamin D supplementation have increased substantially over the last 15 years. Yet, despite the growing number of randomized controlled trials, meta-analyses of these studies have drawn inconsistent conclusions. Many meta-analyses assume that vitamin D is a pharmacological agent, and give scant consideration of it being a nutrient. This limits their potential to detect beneficial effects in participants with vitamin D deficiency. An increasing body of evidence from both observational studies and clinical trials supports the presence of thresholds in vitamin D status below which disease risk increases and vitamin supplementation has beneficial effects. Future supplementation trials which seek to replicate these findings should recruit sufficient numbers of participants with low vitamin D levels, and not give low-dose vitamin D to the placebo group. If the presence of vitamin D thresholds for beneficial effects is confirmed, this would strengthen the need for vitamin D fortification of foods.

## 1. Introduction

**Figure 1:**
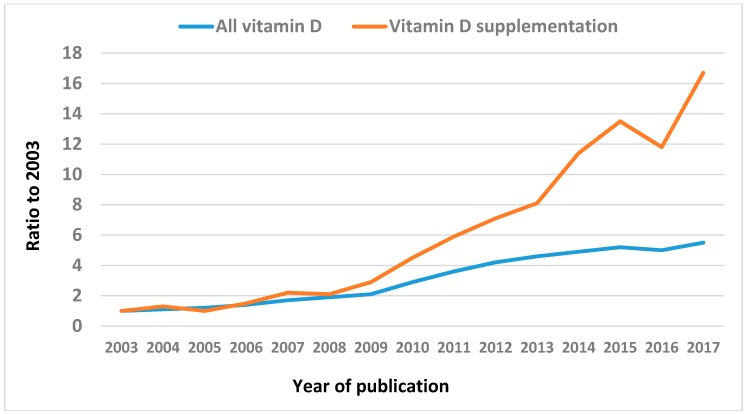
Ratio of number of annual publications (compared to 2003) with vitamin D in the title (all vitamin D), and with vitamin D supplementation or supplement(s) in the title (Pubmed: https://www.ncbi.nlm.nih.gov/pubmed/).

The focus of vitamin D research in recent years has turned to the findings from randomized controlled trials (RCTs) of vitamin D supplementation. Figure 1 shows the more than 15-fold increase over the last 15 years (2003–2017) in the number of publications in PubMed with the term ‘vitamin D supplementation’ in their title. This is much higher than the 5-fold increase for all vitamin D publications. Numbers of publications from vitamin D supplementation trials are expected to continue to grow, based on the >100 vitamin D RCTs currently in the pipeline and registered at ClinicalTrials.gov. In addition, the main findings from several current megatrials, including the US VITAL study [1], the Australian D-Health study [2], and the New Zealand ViDA study [3], are expected to be published over the next 3–4 years [4]. The results from these trials are likely to be influential in deciding whether or not vitamin D supplementation protects against common chronic or acute diseases.

In the meantime, guidelines on the use of vitamin D supplementation have relied on meta-analyses which typically combine the published results of small trials to increase the statistical power of detecting any beneficial effects from vitamin D. The results of these meta-analyses have been inconsistent, with some reporting beneficial effects from vitamin D supplementation [5,6,7,8], and others reporting null effects [9,10,11]. Above these sit the so-called umbrella reviews, which are qualitative reviews of the meta-analyses, and which generally have concluded that vitamin D supplementation has no beneficial effects [12,13]. However, a recent umbrella review did conclude that most RCTs have been carried out in populations that are not vitamin D deficient and, because of this, possible beneficial effects from vitamin D supplementation cannot be excluded [14].

While the major source of vitamin D is endogenous synthesis from sun exposure, a substantial proportion also comes from diet [15]. A common failing of many meta-analyses is that they assume that vitamin D is a pharmacological agent, and give scant consideration to it being a nutrient that can be ingested from food or as a supplement. The implications for the design of clinical trials (from vitamin D being a nutrient) were clearly described by Robert Heaney in an important manuscript published in 2014 [16]. Responses to drugs and nutrients, whether they be biochemical, physiological, or pharmacological, follow a sigmoid curve, with little change in the intended effect of the drug or nutrient at low intake levels until a critical point is reached where the effect increases rapidly in response to increased dose of the drug or nutrient, before plateauing out at higher doses. For most drugs, the intended effect extends over about three orders of magnitude of intake (or a thousandfold), and testing the effect of drug doses typically takes place in the midregion of the sigmoid curve where the line is straight and rapidly rising, resulting in effects that are usually linear. For nutrients, the intended effect usually extends over only one order of magnitude of intake (around 10–20-fold). For example, the usual measure of vitamin D status—circulating 25-hydroxyvitamin D (25(OH)D) concentration—typically ranges from around 25 nmol/L up to 225 nmol/L, and vitamin D intake itself ranges from about 100 international units(IU) per day from diet up to about 2000 IU per day from supplements. Thus, determining the level of circulating 25(OH)D where any benefit starts to increase upwards along the sigmoid curve is necessary if they are to be detected.

Applying this knowledge to a recent clinical trial illustrates how the interpretation of the results can change. The example study found that high-dose vitamin D supplementation (2000 IU/day) did not reduce the number of upper respiratory tract infections compared with low-dose vitamin D (400 IU/day) in Canadian children during winter, leading to the conclusion that routine use of vitamin D supplementation does not prevent these infections [17]. At baseline (September–November), the mean 25(OH)D was 90 nmol/L in the high vitamin D dose arm and 92 nmol/L in the low-dose arm. The standard deviation of about 30 nmol/L indicates that only 5–10 children in each arm were likely to have had baseline 25(OH)D concentrations of <25 nmol/L—the level where beneficial effects were detected in a recent meta-analysis of RCTs of vitamin D supplementation and acute respiratory tract infection [18]. The follow-up mean 25(OHD) of 92 nmol/L in the low-dose vitamin D group, during April–May at the start of the seasonal Spring increase in vitamin D levels, also indicates that only a very small number of children would have had 25(OH)D concentrations of <25 nmol/L during the winter months. Thus, an alternative explanation for the null findings in the Canadian study—findings which imply that vitamin D supplementation is not beneficial against upper respiratory infection—is that very few of the children had vitamin D levels below the threshold to see a benefit from vitamin D supplementation. As such, the Canadian study cannot be considered a proper test of the vitamin D hypothesis, as acknowledged by the authors of this study [19].

A previous review summarized the literature up to 2013 and concluded that the thresholds for beneficial vitamin D status could range from 25(OH)D of 25 nmol/L for bone disease up to 100 nmol/L for cancer [20]. Since then, results from a number of observational and interventional studies have been published; these are discussed below.

## 2. Observational Studies

Meta-analyses of cohort studies have provided some evidence of the location of possible thresholds along the 25(OH)D distribution, below which disease risk increases and benefits from vitamin D supplementation could potentially occur.

A meta-analysis of 32 cohort studies reported a nonlinear association between serum 25(OH)D and the hazard ratio (HR) for all-cause mortality, with an increase in the HR starting in the 25(OH)D range of 75–100 nmol/L, becoming significant in the range of 50 to 74 nmol/L, and increasing to a maximum of 1.9 for people with 25(OH)D < 25 nmol/L [21]. A subsequent meta-analysis of individual participant data (IPD) from seven cohorts with 26,916 participants also found a nonlinear association between 25(OH)D concentrations and the HR for all-cause mortality: the HR was unchanged over the range of 50–125 nmol/L, and increased substantially below 40 nmol/L as 25(OH)D decreased, up to about 2.8 for people with 25(OH)D of <10 nmol/L[22]. Another IPD analysis of 26,018 participants from 8 cohort studies also found curvilinear associations (without any clear threshold) between baseline 25(OH)D concentration and risk of all-cause mortality and cardiovascular mortality [23]. A meta-analysis of 19 cohorts with 65,994 participants found that the risk of cardiovascular disease increased linearly for decreasing circulating 25(OH)D below 60 nmol/L but was unchanged above this value [24].

In contrast, cohort study meta-analyses have not detected nonlinear associations for diabetes [25] and colorectal cancer [26], although a decreasing risk of breast cancer above a 25(OH)D threshold of 67 nmol/L has been observed in postmenopausal (but not premenopausal) women [27]. In addition, a recent large Japanese nested case control study observed a step-down in the hazard ratio of 19% for incidence of all cancer when going from the lowest 25(OH)D quartile to the second; this remained unchanged in higher quartiles, suggesting a threshold effect at about 40–45 nmol/L [28].

Overall, meta-analyses of cohort studies suggest there is a threshold effect of vitamin D status on the risk of some chronic diseases, particularly for all-cause mortality and cardiovascular disease, at circulating 25(OH)D concentrations in the range of 40–75 nmol/L. This broad range for thresholds reported in observational studies could reflect differences in the quality of 25(OH)D assays, with some reading high and others low, thus making the actual cut-point unclear [29].

## 3. Interventional Studies

Evidence is starting to accumulate from clinical trials of threshold effects from vitamin D supplementation for preventing several clinical and physiological outcomes. These reports are a mixture of meta-analyses and individual RCTs, with several published in the last year (Table 1).

### 3.1. Meta-Analyses

The earliest is a Cochrane meta-analysis which found that vitamin D supplementation, compared with placebo, reduced the risk of falls in four studies that selected people with lower vitamin D levels [30]. The four studies had cut-points of <30, <50, <78, and <60 nmol/L [31,32,33,34]. The 30% reduction in the risk of falls in these studies (risk ratio = 0.70; 95% confidence interval (CI): 0.56–0.87) was significantly lower than in the other nine studies that did not select participants based on vitamin D status (risk ratio = 1.00; 95% CI: 0.93–1.07; *P*_interaction_ < 0.01).

An IPD meta-analysis of 25 trials found that vitamin D supplementation, compared with placebo, significantly reduced the odds of having an acute respiratory infection in participants with baseline 25(OH)D levels of <25 nmol/L (odds ratio = 0.58; 95% CI: 0.40–0.82), equivalent to a 26% reduction in risk; this was more than in participants with levels ≥25 nmol/L (odds ratio = 0.89; 95% CI: 0.77–1.04; *P*_interaction_ = 0.01) where the risk reduction was a nonsignificant 6% [18].

A further IPD meta-analysis of seven trials, led by the same group [35], reported that vitamin D supplementation, when compared with placebo, reduced the incidence rate of exacerbations in asthma patients who had baseline 25(OH)D concentrations of <25 nmol/L by 67% (rate ratio = 0.33; 95% CI: 0.11–0.98), but not in those with baseline levels ≥25 nmol/L (rate ratio = 0.77; 95% CI: 0.58–1.03). The *p*-value for the interaction was not significant, and the rate ratio combining all participants was significant (rate ratio = 0.74, 95% CI: 0.56–0.97). However, given the relatively small number of participants (*n* = 856), more studies are required to determine whether the protective effect of vitamin D supplementation against asthma exacerbations varies with vitamin D status.

### 3.2. Individual Trials

Recent results from individual studies also provide evidence for the effect of vitamin D supplementation being greater in people with vitamin D deficiency.

A trial in patients with chronic obstructive pulmonary disease in Belgium found that vitamin D supplementation given orally as bolus 100,000 IU doses every 4 weeks for one year reduced the incidence rate of exacerbations from this condition by 43% in patients with baseline 25(OH)D concentrations of <25 nmol/L—a greater reduction than in those with higher 25(OH)D levels (*P*_interaction_ = 0.027) [36].

A trial from Austria found that vitamin D supplementation—a 540,000 IU bolus followed by 90,000 IU monthly for 6 months given orally or by nasogastric tube—reduced hospital mortality in patients admitted to intensive care units by 38% after 6 months in those who had baseline 25(OH)D levels of ≤30 nmol/L (HR = 0.56; 95% CI: 0.35–0.90) but not in those above this level (HR = 1.12; 95% CI: 0.72–1.77; *P*_interaction_ = 0.04) [37].

Findings from the recently completed Vitamin D Assessment (ViDA) study carried out in New Zealand, which gave an oral bolus vitamin D dose of 100,000 IU/month [3], show significant interactions in the effect of vitamin D supplementation on some outcomes with regard to baseline 25(OH)D level. There was greater attenuation of bone mineral density loss in the spine and femoral neck after 2 years in people with 25(OH)D ≤ 30 nmol/L compared with in those above this level (*P*_interaction_ = 0.04) [38]. The reduced decline in bone mineral density (2% over 2 years) from vitamin D was small relative to other more powerful anti-osteoporotic drugs, but vitamin D supplementation may be important if targeted at populations known to have low 25(OH)D levels such as the elderly living in institutions [39]. The beneficial effect in participants with low 25(OH)D levels is consistent with the increased bone mineral density seen in infants born in winter to mothers given vitamin D supplements during pregnancy, but not in births in other seasons [40].

Interactions in the effect of vitamin D supplementation by baseline vitamin D status were seen also for several measures of arterial function in the ViDA study [41]. Greater reductions in arterial waveform parameters, such as augmentation index and pulse wave velocity, were seen after 12 months of supplementation in participants with baseline 25(OH)D < 50 nmol/L compared with those above this cut-point (all *P*_interaction_ < 0.05). The clinical significance of these findings is unclear at the moment until further research quantifies the role of these parameters in predicting cardiovascular disease. In addition, vitamin D supplementation for one year (compared with placebo) increased forced expiratory volume in 1 s (FEV_1_), measured by spirometry, more in participants who had ever smoked tobacco and had 25(OH)D levels of <50 nmol/L (by 109 mL or 5%) than in those who had ever smoked and had higher vitamin D levels (*P*_interaction_ = 0.048) [42]. Changes in FEV_1_ of >100 mL are clinically significant [43].

Overall, these trials reported threshold effects from vitamin D supplementation that are beneficial for people with 25(OH)D concentrations of <25–30 nmol/L, and which may extend up to 50 nmol/L for measures such as arterial function and lung function. Their results indicate that if future research aims to determine with certainty whether vitamin D supplements are beneficial in people with vitamin D deficiency, researchers need to recruit sufficient participants with low 25(OH)D concentrations, and not give low-dose vitamin D to the control arm, as was done in the Canadian winter study [17]. This is because there is an inverse association between baseline 25(OH)D levels and the increase in 25(OH)D response to supplementation [44,45], such that even low vitamin D doses, when given to vitamin-D-deficient people, have the potential to increase 25(OH)D levels above a deficiency threshold of 25 nmol/L.

The need for future supplementation trials to recruit people who are vitamin D deficient, and not give any dose of vitamin D supplement to the placebo arm, does raise serious practical and ethical issues. One strategy around this is to recruit participants from populations likely to have low 25(OH)D concentrations (e.g., those who are older, institutionalized, or with increased skin pigmentation), but not measure baseline 25(OH)D level. By not identifying those who are deficient at baseline, the ethical requirement to provide vitamin D supplements is avoided. Instead, blood samples can be collected at baseline and stored for later measurement of 25(OH)D so that vitamin-D-deficient participants can be identified after follow-up has been completed. This strategy has been used by some of the current mega-trials [1,2,3] and has been justified on the basis of the current state of equipoise regarding the lack of knowledge about the nonskeletal effects of vitamin D. The alternative—of excluding vitamin-D-deficient people or giving low vitamin D doses to the placebo group—is to continue to fail to detect possible beneficial effects from vitamin D supplementation as reported in some recent studies [17,46].

Results from further RCTs may clarify whether the threshold for beneficial effects from vitamin D supplementation varies between different disease outcomes, as previously proposed [20]. Such trials will need to ensure they use 25(OH)D assays that are standardized to current gold standard reference methods to avoid confusion about the level of any detected thresholds [29].

## 4. Daily versus Intermittent Vitamin D Supplementation

In contrast, beneficial effects from vitamin D supplementation have not been observed for some outcomes in vitamin-D-deficient people. For example, the ViDA study did not see a reduction in the risk of cardiovascular disease, falls, or fractures in the vitamin D arm among participants with 25(OH)D concentrations of <50 nmo/L [47,48]. A possible explanation for the lack of vitamin D effect against these outcomes in vitamin-D-deficient people was the use of a monthly bolus dose of vitamin D in the ViDA study. Even though a sufficiently high vitamin D dose was given in the ViDA study to increase mean 25(OH)D levels in the treatment arm above 100 nmol/L, it is possible that the short half-life of vitamin D, which is more easily transported into cells than 25(OH)D, resulted in short-lived peaks in intracellular vitamin D followed by longer periods of insufficient vitamin D [49,50].

Some evidence in support for this comes from the meta-analysis of respiratory infection which found that daily or weekly vitamin D supplementation resulted in a stronger reduction in the odds of respiratory infection than bolus dosing (*P*_interaction_ = 0.05) [18]. However, for several outcomes, such as those seen in the ViDA study for bone density and arterial and lung function [38,41,42], and in the trials of COPD exacerbations [36] and mortality in patients admitted to intensive care units [37], the evidence shows that bolus dosing is beneficial among people with low vitamin D levels. Further evidence comparing the effects of daily versus bolus dosing will emerge within the next 2–3 years from comparisons between the VITAL study, which gave daily (2000 IU) dosing [1], and the D-Health study, which gave the same size dose but monthly (60,000 IU) [2]. The measurement of circulating cholecalciferol has the potential to clarify any differences in effect between daily and bolus dosing [50].

## 5. Public Health Implications

If evidence continues to accumulate of beneficial effects from vitamin D supplementation that are confined to, or maximal in, people with low 25(OH)D levels, this will have implications for public health strategies for preventing vitamin D deficiency. Traditionally, the two approaches for prevention have been the high-risk approach and the population approach [51]. The former would involve screening people for vitamin D deficiency using the 25(OH)D test, and then providing vitamin D supplements to those who are deficient, with the 25(OH)D threshold for deficiency determined by the ongoing research. Such an approach would have a high cost–benefit ratio if the 25(OH)D threshold was closer to 25 nmol/L than to 50 nmol/L, as only a small proportion of the population would benefit from vitamin D supplements, while a much larger proportion would need to be screened to detect those who are deficient.

The cost–benefit ratio would decline for population groups with low 25(OH)D levels, such as South Asians [52], in whom the prevalence of vitamin D deficiency is high and the number needed to screen to detect one case of vitamin D deficiency would be low. In such communities, a population prevention strategy of vitamin D supplementation without any 25(OH)D screening could be considered, as vitamin D is very cheap and has no side effects of clinical significance when given in recommended doses [53,54].

A more effective approach to preventing vitamin D deficiency when it occurs in only a small proportion of the community would be the population approach of vitamin D fortification, as it avoids the large costs from 25(OH)D screening of the whole population to detect and supplement the minority with vitamin D deficiency. Adding small amounts of vitamin D to commonly eaten foods, at low cost, would shift the distribution of 25(OH)D in the whole population to the right and minimize the proportion who are vitamin D deficient [55]. Modelling based on national food consumption data and the level of achievable vitamin D fortification would need to be carried out for individual countries to determine the effectiveness of this approach in shifting the 25(OH)D distribution to the right. Recent analyses indicate that a dietary vitamin D intake of 26 μg/day would be required to ensure that 97.5% of adults had 25(OH)D levels above 50 nmol/L [56]; this is unlikely to be achieved with fortification by itself and probably requires some vitamin D supplementation. Moreover, the daily requirement for vitamin D varies between sections of the population, being highest in older adults [57]. However, the need for such modelling research would increase if future RCTs confirm 25(OH)D thresholds for beneficial effects from vitamin D supplementation.

## 6. Conclusions

An increasing body of evidence from both observational studies and clinical trials supports the presence of thresholds in vitamin D status below which disease risk increases and vitamin supplementation has beneficial effects. Further trials are required to determine whether or not the findings summarized in Table 1 are false-positive results due to chance. Future supplementation trials which seek to replicate these findings should recruit sufficient numbers of participants with low vitamin D levels (all with 25(OH)D < 50 nmol/L, and a good proportion with <25 nmol/L), not give low-dose vitamin D to the placebo group, and include the measurement of circulating cholecalciferol (at least in a subgroup). If the presence of vitamin D thresholds for beneficial effects is confirmed, this would strengthen the need for vitamin D fortification of foods.

## Figures and Tables

**Table 1:** Summary of meta-analyses of randomized controlled trials of vitamin D supplementation and individual trials which have shown a beneficial effect in participants with low vitamin D status.

Study	Design	Outcome	Baseline 25(OH)D Subgroup: No. Vitamin D/No. Placebo	Results for Vitamin D Subgroups Measure of Effect (95% CI)	*p*-Value for Interaction
**Meta-Analyses**					
Gillespie 2012 [30]	Meta-analysis of 13 RCTs of people living in the community	Falls	Study selected for low vitamin D: Yes: 405/399 No: 12,636/13,307	RR = 0.70 (0.56, 0.87) RR = 1.00 (0.93, 1.07)	<0.01
Martineau 2017 [18]	IPD meta-analysis of 25 RCTs of people from a range of settings	Acute respiratory infection	25(OH)D < 25 nmol/L: 289/249 25(OH)D ≥ 25 nmol/L: 1995/1639	OR = 0.58 (0.40 to 0.82) OR = 0.89 (0.77 to 1.04)	0.01
Jolliffe 2017 [35]	IPD meta-analysis of 7 RCTs of asthma patients	Asthma exacerbations	<25 nmol/L: 92 patients in 3 trials ≥25 nmol/L: 764 patients in 6 trials	IRR = 0.33 (0.11–0.98) IRR = 0.77 (0.58–1.03)	0.25
**Individual Trials**					
Lehouck 2012 [36]	Single RCT of COPD patients	COPD exacerbations	25(OH)D < 25 nmol/L: 15/15 25(OH)D ≥ 25 nmol/L: 76/76	Lower IRR = 0.57 (0.33 to 0.98) in patients with 25(OH)D < 25 vs. ≥25 nmol/L.	0.027
Amrein 2014 [37]	Single RCT of patients admitted to intensive care units	Mortality (in hospital)	25(OH)D ≤ 30 nmol/L: 102/98 25(OH)D > 30 nmol/L: 136/139	HR = 0.56 (0.35–0.90) HR = 1.12 (0.72–1.77)	0.04
Reid 2017 [38]	Single RCT of community resident adults	Bone mineral density (change over 2 years)	25(OH)D ≤ 30 nmol/L: 25/21 25(OH)D > 30 nmol/L: 179/185	Greater attenuation of spine and femoral neck BMD loss in people with 25(OH)D ≤ 30 vs. >30 nmol/L.	0.04
Sluyter 2017 [41]	Single RCT of community resident adults	Arterial function (change over 1 year)	25(OH)D < 50 nmol/L: 71/79 25(OH)D ≥ 50 nmol/L: 122/108	Greater reduction in several arterial waveform parameters (e.g., augmentation index, pulse wave velocity) in people with 25(OH)D < 50 vs. ≥50 nmol/L.	<0.05
Sluyter 2018 [42]	Single RCT of community resident adults	Lung function (change over 1 year)	Ever smoked tobacco: 25(OH)D < 50 nmol/L: 26/28 25(OH)D ≥ 50 nmol/L: 78/85	Greater increase in FEV_1_ in ever smokers with 25(OH)D < 50 vs. ≥50 nmol/L.	0.048

BMD = bone mineral density; CI = confidence interval; COPD = chronic obstructive pulmonary disease; FEV_1_ = forced expiratory volume in 1 second; HR = hazard ratio; IPD = individual patient data; IRR = incidence rate ratio; OR = odds ratio; RCT = randomized controlled trial; RR = risk ratio.
